# When the levee breaks: a practical guide to sketching algorithms for processing the flood of genomic data

**DOI:** 10.1186/s13059-019-1809-x

**Published:** 2019-09-13

**Authors:** Will P. M. Rowe

**Affiliations:** 1 Institute of Microbiology and Infection, School of Biosciences, University of Birmingham, Birmingham, B15 2TT UK; 2 Scientific Computing Department, The Hartree Centre, STFC Daresbury Laboratory, Warrington, WA4 4AD UK

## Abstract

Considerable advances in genomics over the past decade have resulted in vast amounts of data being generated and deposited in global archives. The growth of these archives exceeds our ability to process their content, leading to significant analysis bottlenecks. Sketching algorithms produce small, approximate summaries of data and have shown great utility in tackling this flood of genomic data, while using minimal compute resources. This article reviews the current state of the field, focusing on how the algorithms work and how genomicists can utilize them effectively. References to interactive workbooks for explaining concepts and demonstrating workflows are included at https://github.com/will-rowe/genome-sketching.

## Introduction

To gain biological insight from genomic data, a genomicist must design experiments, run bioinformatic software and evaluate the results. This process is repeated, refined or augmented as new insight is gained. Typically, given the size of genomic data, this process is performed using high performance computing (HPC) in the form of local compute clusters, high-memory servers or cloud services. HPC offers fast disk-access, a large number of processors and high-memory. But HPC resources are in demand, with researchers queuing to use them, or having limited funds to access them. Worse yet, what happens when an experiment has too much data to be realistically stored or analyzed using these resources? Similarly, given the advent of real-time sequencing technologies, what if researchers want to ask questions of data as they are being generated or cannot wait for HPC to become available?

As genomics continues to thrive, from basic research through to personalized genome services, data continue to flood into genome archives and databases. One of the many consequences of this has been that genomicists now have a wealth of data to choose from when they design their experiments. This requires sampling considerations to be made, such as the quantity, quality and distribution of data. In an ideal world, most genomicists would elect to include all available data but this is growing harder to achieve as the amount of data drives up runtimes and costs.

In response to being unable to analyze *all the things*, genomicists are turning to analytics solutions from the wider data science field in order to process data quickly and efficiently [1–4]. In particular, the model of streaming data processing is proving incredibly effective in minimizing the resource usage of genomic analysis. Rather than capturing, sorting and indexing every piece of data, streaming data processing instead quickly looks at each piece of data as it is received and uses this information to summarize the current state. Once a piece of data has been processed it is no longer accessible; only the overall summary is kept [5]. This summary is termed a sketch and serves as an approximation of the data that was processed (Table 1).

**Table 1:** Glossary of terms

Term	Definition
Bit-pattern observable	The run of 0 s in a binary string
Bit vector	An array data structure that holds bits
Canonical k-mer	The smallest hash value between a k-mer and its reverse complement
Hash function	A function that takes input data of arbitrary size and maps it to a bit string that is of fixed size and typically smaller than the input
Jaccard similarity	A similarity measure defined as the intersection of sets, divided by their union
K-mer decomposition	The process of extracting all sub-sequences of length k from a sequence
Minimizer	The smallest hash value in a set
Multiset	A set that allows for multiple instances of each of its elements (i.e. element frequency)
Register	A quickly accessible bit vector used to hold information
Sketch	A compact data structure that approximates a data set
Stochastic averaging	A process used to reduce the variance of an estimator

Sketch data structures are relatively small so fit entirely in memory; they need only a single pass of the data and you can use a sketch before the underlying data stream has terminated [6]. This makes sketching faster and more efficient than high latency alternatives; you do not have to store an entire data stream and you can analyze data in real-time [4, 7]. Another major advantage of sketch data structures is that they can be used to estimate averages; which in the case of genomics can be used to approximate the similarity of genomes without using all the sequence data [1].

The next section outlines some properties of sketches and how they can be used to approximate the underlying data. In subsequent sections, the core sketching algorithms are described, detailing their uses, advantages, variants and current implementations for genomics. Interactive workbooks to demonstrate key concepts and workflows that utilize sketching to tackle real-world genomics problems are provided (see **“**Availability of data and material” section) [8].

## What is sketching

The concept of data sketching has been around for several decades, originating with probabilistic counting algorithms that can estimate the number of distinct elements within a dataset on disk [9]. Sketching has more recently been used to summarize data streams; first applications provided an ephemeral overview of data and more recently have offered persistent summaries of data streams [10].

Put simply, sketching is the process of generating an approximate, compact summary of data. A sketch supports a set of predetermined query and update operations, which are used to approximate the original data. Compared with non-probabilistic algorithms, sketching requires less memory and has constant query time [5].

To be considered a sketching algorithm, several requirements must be satisfied. Cormode et al. [6] state that sketch updates must be consistent, irrespective of sketch history or the current sketch state. The sketching process results in a probabilistic data structure (the sketch) that is a linear transform of the input data. Sketches typically implement four key methods: create, update, merge and query. The ability to update and merge means parallelization is often achievable.

It should be stressed that sketching is not sampling. Although both allow for data to be summarized, sampling does not allow certain questions to be asked of the data, e.g. set membership. Sketching can yield better estimates than sampling. Standard error of a sample of size *s* is $\frac{1}{\sqrt{s}}$, whereas sketches of size *s* can guarantee error that is proportional to $\frac{1}{s}$ [5].

Sketching effectively compresses data, resulting in low memory requirements, queries in linear-time and reduced bandwidth requirements in distributed systems. Consequently, sketching has applications in data analytics, signal processing and dimensionality reduction. So if you can accept an approximate answer and need it quickly, sketching algorithms fit the bill. Which particular algorithm to use depends on the nature of the question you want to ask.

## Sketching algorithms and implementations

### Set similarity with MinHash

#### Set similarity

Say we wish to compare two music collections, each containing 100 records. Each collection is a set and we can use Jaccard similarity, defined as the size of the intersection of two sets, divided by the size of their union, to measure their similarity. If our two collections have 60 records in common, then the Jaccard similarity is 60 divided by the number of distinct records 140, giving 0.429.

Jaccard similarity is regularly used in data science, for tasks such as document aggregation and duplicate detection [11]. Document similarity can be based simply on the number of shared words (a “bag of words” model), but to also take into account document structure, it may be better to represent the document as a set of overlapping groups of words (termed “n-grams”). For instance, the following sentence “We come from the land of the ice and snow” can be broken into five n-grams (where *n* = 6): “We come from the land of”, “come from the land of the”, “from the land of the ice”, “the land of the ice and” and “land of the ice and snow”. Now, compare that with another sentence: “The hammer of the gods will drive our ships to new land”. Both of these sentences share the words “of”, “the” and “land” but not in the same n-gram context, so they do not have any similarity when you account for document structure. The Jaccard similarity is 0.176 using a bag of words model, but 0 using a bag of n-grams model (where *n* = 6), which retains some structure.

Looking at this example, you can tell it is a bit impractical to use the groups of words as they are; a 10-word sentence turns into five 6-word groups. Instead, we can *hash* these groups. Hash functions take large input data of arbitrary size and map it to a bit string of fixed size (called a hash value). So, each of these groups of words would be mapped to a hash value, and documents are compared by calculating the Jaccard similarity between the sets of hash values.

Even when using sets of hash values, you can see that Jaccard similarity calculations will get harder to compute as the sets get bigger, or the number of sets increases. In fact, in a worse-case scenario, pairwise similarity calculations scale quadratically in terms of time and space complexity. To perform set similarity queries efficiently on large datasets, we could accept approximate answers and look to sketching algorithms.

#### MinHash algorithm

The MinHash algorithm generates a sketch that is designed to allow scalable approximation of the Jaccard similarity between sets. It was originally developed for detection of near-duplicate web pages and images [12].

MinHash, as with other sketching algorithms, relies on hash functions. The hash functions used in sketching algorithms should be uniform and deterministic, i.e. input values should map evenly across an output range and a given input should always produce the same hash value. By applying a hash function to every element in a set and sorting the set by the resulting hash values, a pseudo-random permutation of the set is achieved. Taking this idea a step further, if the same hash function is applied to two sets, the chance of both sets having the same minimal hash value is going to be equal to the ratio of the number of common elements to the size of the union, i.e. the Jaccard similarity. Broder [12, 13] first demonstrated this concept in his seminal work on MinHash.

The MinHash sketch data structure is a vector of hash values, plus some extra information describing how the sketch was made (e.g. which hash function). There are several different MinHash “flavors”, such as k-hash functions (KHFs) sketch, k-minimum values (KMVs) sketch and the k-partition sketch [13–19].

For now, let us focus on MinHash KHF sketches as this is the classic example of MinHash. As the name suggests, this flavor of MinHash uses *K* hash functions to generate *K* permutations of the set. For each permutation, the minimum value is added to the sketch and all other values are ignored (Algorithm 1).

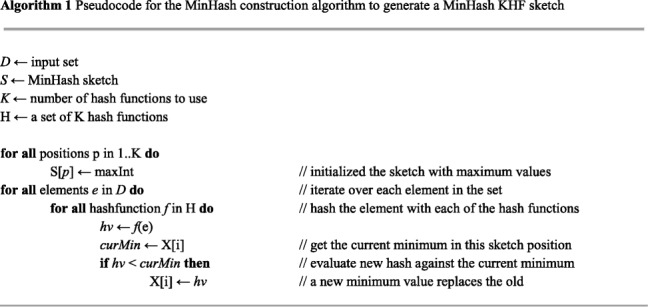



To estimate the Jaccard similarity of two sets using their KMV sketches, we compare the values in each position of the sketches and increment a counter if they match. The counter is then divided by sketch length, yielding the Jaccard similarity estimate. As this is probabilistic, we can increase the accuracy by increasing the number of random permutations sampled; the longer the sketch, the better the Jaccard similarity estimate. However, as having lots of unique hash functions is expensive, KHF sketching often uses K min-wise, independent permutations and a strong universal hash function that maps one hash value onto several other hash values.

An alternative to this approach is to have just one hash function, instead sampling K minimum values from the set. This is KMV sketching (also known as bottom-K sketching) and has the advantage that the sketch only needs to be updated if a new hash value is encountered that is smaller than the maximum value currently in the sketch. This means that KMV sketches can be straightforward to implement using a priority queue, allowing quick evaluation of a new hash value against the largest value currently in the queue. Another advantage with KMV sketching is that accuracy increases linearly with K; so for a set of N elements, when K ≥ N, accuracy = 1 as all elements will be in the sketch. This accuracy scaling is not guaranteed by a KHF sketch.

It is important to remember that when comparing MinHash sketches, they must have been constructed using the same MinHash algorithm, as well as with the same hash functions so that shared elements will yield the same hash values.

#### MinHash implementations for genomics

**Fig. 1:**
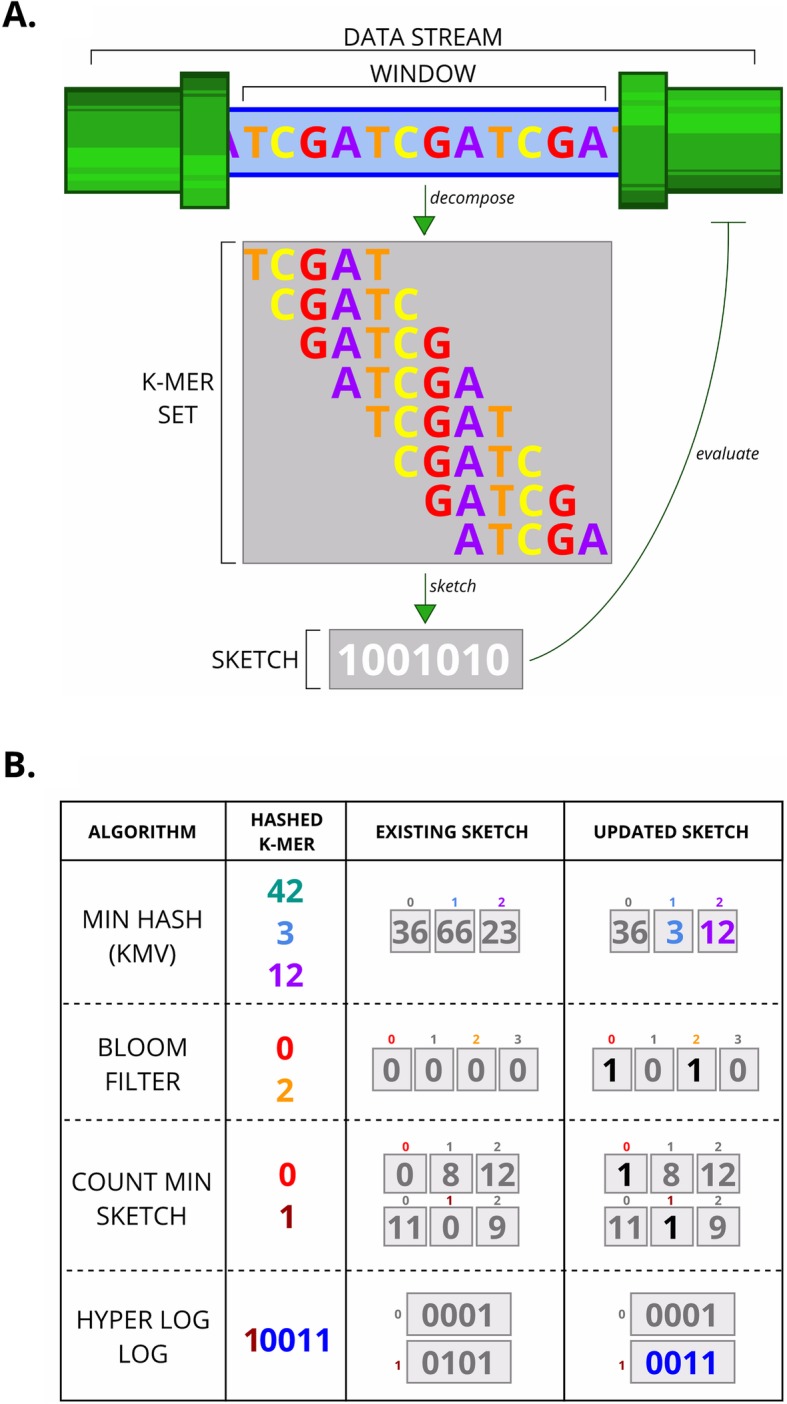
**a** Sketching applied to a genomic data stream. The genomic data stream is viewed via a window; the window size may be equivalent to the length of a sequence read, a genome or some other arbitrary length. The sequence within the window is decomposed into a set of constituent k-mers; each k-mer can be evaluated against its reverse complement to keep only the canonical k-mer. As k-mers are generated, they are sketched and the sketch data structure may be updated. The sketch can be evaluated and allow feedback to the data stream process. **b** Common sketching algorithms applied to a single k-mer from a set, using example parameters. MinHash KHF: the k-mer is hashed by three functions, giving three values (*green*, *blue*, *purple*). The number of hash functions corresponds to the length of the sketch. Each value is evaluated against the corresponding position in the sketch; i.e. *green* compared against the first value, *blue* against the second, and *purple* against the third. The sketch updates with any new minimum; e.g. the *blue* value is smaller than the existing one in this position (3 < 66), so replaces it. Bloom filter: the k-mer is hashed by two functions; giving two values (*red* and *orange*). The output range of the hash functions corresponds to the length of the sketch, here 0–3. The hash values are used to set bits to 1 at the corresponding positions. CountMin sketch: the k-mer is hashed by two functions; giving two values (*red* and *brown*). The number of functions corresponds to a row in the sketch, here 0 or 1, and the output range of the functions corresponds to the length of the rows, here 0–2. So the first hash value (*red*) gives matrix position 0,0 and the second gives 1,1. The counters held at these positions in the matrix are incremented. HyperLogLog: the k-mer is hashed by one function; giving a single value (10011). The prefix (*brown*) corresponds to a register, and the suffix (*blue*) corresponds to the bit-pattern observable. The suffix is compared to the existing value in register 1, is found to have more leading zeros and so replaces the existing value in register 1

To apply MinHash to a genomic data stream, we rely on the bioinformatic workhorse of k-mer decomposition. This involves breaking down a sequence into a set of overlapping subsequences of length *k*, termed k-mers, equivalent to the word groups in our earlier example (Fig. 1a). We do not need to differentiate between k-mers and their reverse complement, so we hash them both and keep only the smaller of the two hash values (the canonical k-mer).

Some of the earliest implementations of MinHash for genomic applications were MHAP and MC-MinH, which applied MinHash sketching to genome assembly and metagenome sequence clustering, respectively [20, 21]. These ideas were taken further with MASH, where the authors demonstrated that KMV MinHash sketches facilitate fast, approximate sequence similarity searches and provide an efficient method of sequence compression [1]. The MASH authors extended MinHash to incorporate a pairwise mutation distance and *P* value significance test. MASH is able to accurately cluster genome and metagenome data, as well as perform real-time sequence database search. Since MASH, other multipurpose and well-documented MinHash libraries for genomics have been developed, such as sourmash, Finch and BBSketch (part of BBMap) [22–24].

As well as these libraries, several bioinformatic programs utilize the MinHash algorithm to provide fast and efficient answers for common genomics workflows (Table 2). These include GROOT, which uses KHF sketching for variant detection in metagenome samples [25], mashtree, which uses KMV sketching for phylogenetic tree construction [26], and MashMap, which uses KMV sketching for long read alignment [27, 41]. MashMap now also uses minimizers, which is a concept closely related to MinHash. By sliding a window across a sequence and decomposing windows to k-mers, the smallest hashed k-mer is the minimizer for that window. The minimizers from each window make up the sketch. Minimizers were proposed by Roberts et al. [42] as a sequence compression method, and have been popularized by the MiniMap read aligner [28, 43].

**Table 2:** Examples of bioinformatic software utilizing sketching algorithms

Software	Purpose	Sketching algorithm
GROOT [25]	Variant detection in metagenomes	MinHash (KHF)
mashtree [26]	Phylogenetic tree construction	MinHash (KMV)
MashMap [27]	Long read alignment	Minimizer/MinHash (KMV)
MASH [1]	Sequence analysis	MinHash (KMV)
sourmash [22]	Sequence analysis	MinHash (KMV)
finch [23]	Sequence analysis	MinHash (KMV)
MiniMap2 [28]	Read alignment	Minimizer
ABySS [29]	Genome assembly	Bloom filter
Lighter [30]	Sequencing error correction	Bloom filter
BIGSI [31]	Sequence index and search	Bloom filter
khmer [32]	Sequence analysis	Count-Min sketch
FastEtch [33]	Genome assembly	Count-Min sketch
dashing [2]	Sequence analysis	HyperLogLog
krakenUniq [34]	Metagenome classification	HyperLogLog
HULK [4]	Sequence analysis	Histosketch
ntCard [35]	Sequence analysis	ntCard
BBsketch [24]	Sequence analysis	MinHash
MHAP [21]	Genome assembly	MinHash
MC-MinH [20]	Sequence clustering	MinHash
KmerGenie [36]	Sequence analysis	Count-Min sketch variant
Squeakr [37]	Sequence analysis	Counting Quotient Filter
Mantis [38]	Sequence index and search	Counting Quotient Filter
kssd [39]	Sequence analysis	K-mer Substring Space Decomposition

An up-to-date list is provided in [40]

#### Considerations and variations

MinHash is an efficient way to estimate Jaccard similarity between sets. It was described above that Jaccard similarity is based on the union and intersection of sets, and that in genomics we consider k-mers as set elements in order to account for sequence structure. However, Jaccard similarity does not take into account element frequency within a set (referred to as a multiset). In genomics, we may want to include k-mer frequency in our similarity estimates, particularly when dealing with metagenomes. Several MinHash implementations have provision for multisets. For example, sourmash keeps a record of k-mer frequencies if requested (−-*track-abundance*) [22], and Finch has a clever *over-sketching* method, which creates a parent sketch with tracked k-mer abundance which is used to populate a smaller child sketch with dynamic filtering [23].

Another consideration is how to handle sets of different size; MinHash performance degrades with increasing difference in set size [44]; for instance, using MinHash to find a genome within a metagenome. One solution is to combine MinHash with other data structures and utilize a containment index; set intersection is divided by the size of one of the sets to normalize for imbalance. This approach is now offered by sourmash, Finch and MASH [22, 23, 45]. Sourmash also features several interesting alternatives, such as k-mer binning and greedy partitioning (see *lca* and *gather* sub-commands) [22].

As well as the MinHash libraries discussed already, several recent algorithm variations deserve mentioning. BinDash [46] is an implementation of binwise densified MinHash, offering improved speed, precision and compression performance over KMV sketching implementations. Order MinHash [47] is a variant that considers the relative order of k-mers within sequences, enabling estimation of edit distance between sequences. HyperMinHash and b-Bit MinHash are MinHash variants that offer compressed sketches, and are typically a trade-off between accuracy, speed and storage cost [17, 48].

### Set membership with Bloom filters

#### Set membership

From our earlier example of set similarity, we know that we have similar taste in music. Let us say that you now want to listen to the records in my collection that you have not got in yours. This is a set membership query and, in a worst-case scenario, will require a loop through your collection for each record in my collection.

This worst-case scenario can be improved upon. For example our record collections could (or should!) be sorted alphabetically, meaning if I had “AC/DC - Powerage” and went through all the As in your collection without finding it, I would not need to continue looking and could play the record. We could also improve our search by remembering the entirety of your collection, bypassing the need to loop through your collection for each record in mine.

However, sorting sets can take time and memorizing sets can be difficult or impossible; our example would not scale well if we had millions of records. Fortunately, sketching algorithms allow us to approximate set membership queries and return answers quickly.

#### Bloom filter algorithm

The Bloom filter algorithm produces a sketch for set membership queries; telling us if an element is possibly in a set, or if it is definitely not in the set (i.e. it allows false positives but no false negatives). Once you add an element to a Bloom filter, a subsequent query using the same element will tell you that you have probably seen it before. Elements cannot be removed from a Bloom filter and the more elements you add, the larger the probability of false positives [49].

The Bloom filter algorithm uses hash functions to populate a bit vector (the sketch), which is essentially a row of bits that can be set to 0 or 1 (Algorithm 2). To begin, all bits are set to 0. When adding an element to the sketch, multiple hash functions are used to map the element to several positions in the sketch. At each mapped position, the bit is changed to a 1 if not already set, and cannot be changed back during the life of the sketch.

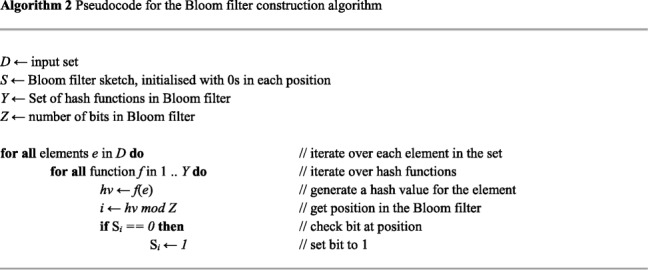



To perform a set membership query, the query is hashed using the same functions used to create the Bloom filter. Each of the returned sketch positions is checked; if all bits are set to 1 then the element has probably been seen before. If one or more bits are set to 0, the element has definitely not been seen before. This is thanks to the deterministic property of the hash functions, meaning that an element will always be hashed to the same value.

To calibrate a Bloom filter, the false-positive rate is inversely proportional to the sketch length (the number of bits). The longer the sketch, the greater the number of possible hash values and the lower the chance of different elements hashing to the same value (a false positive); this is known as a hash collision. The more hash functions a Bloom filter uses the slower it will be, but using too few functions or too many will result in more false positives. A simple calculation computes the optimal number of hash functions for a given size of Bloom filter, but requires knowing an estimate of the number of distinct elements (Eq. 1).

The optimal number of hash functions (*k*) to minimize the false-positive rate for a Bloom filter of size m, for an estimated number of distinct elements (*n*)

1 $$ k=\frac{m}{n}\ \mathit{\ln}\ 2 $$

To decide how long to make the sketch and how many hash functions to use, optimization calculations can be performed to parameterize the sketch to give approximations within specified error bounds [50].

#### Bloom filter implementations for genomics

To apply a Bloom filter to a genomic data stream, we again use k-mer decomposition. The canonical form of each k-mer is passed through a Bloom filter; if the k-mer has not been seen before then it is added to the sketch (Fig. 1b). This can have several uses, such as approximating k-mer counts (using an additional hash table to track counts), or excluding unique k-mers from analyzes.

Although Bloom filters were first used in bioinformatics around 2007, one of the first genomics applications was BFCounter in 2011 [51]. BFCounter used a Bloom filter to track k-mer counts; it used this sketch to give an approximation of k-mer abundance, or generated exact counts by combining the sketch with a hash table and performing a second pass of the data.

K-mer counting is a very common component in many genomic processes, such as sequence assembly, compression and error correction. Software that utilize Bloom filters for these processes include Minia, ABySS, Xander and dnaasm for assembly [44, 52–54], Quip for compression [55], and Musket, BLESS and Lighter for error correction [45, 56, 57]. Bloom filters are also used in conjunction with other sketching algorithms, such as by MASH to prevent singleton k-mers (which often arise from sequencing error) from being added to the MinHash sketch [1].

#### Considerations and variations

Although Bloom filters offer many performance advantages over non-sketching algorithms for set membership queries, as illustrated by their ubiquity in data science and genomics, they have several shortcomings which must be considered prior to their use. Limitations include the inability to remove elements, dynamically resize the sketch or count the number of occurrences of each item. Several variants of the Bloom filter algorithm aim to improve on these shortcomings, including counting, multistage and spectral Bloom filters [3, 58], Cuckoo filters [59] and counting quotient filters [60].

In addition to these variants, Bloom filters have been used as building blocks in several algorithms for genomic indexing problems. One example is sequence bloom trees, which are a hierarchy of compressed Bloom filters with each one containing a subset of the items being indexed [61]. Sequence bloom trees have been combined with MinHash to allow disk-based search of sketches [22].

A recent indexing algorithm, the Bit-sliced Genomic Signature Index (BIGSI) [31], utilizes a set of indexed Bloom filters for real-time sequence search. BIGSI improves upon other methods, including sequence bloom trees, which suffer from a performance drop when indexing diverse sequences (i.e. a scaling dependence on the total number of k-mers in the union of sequences being indexed). To create a BIGSI index, each sequence (e.g. genome assembly) is decomposed to k-mers, hashed *N* times and sketched using a Bloom filter; each Bloom filter is stored as a column in a matrix (the index). To query the index a k-mer is hashed *N* times to give *N* row indices; the corresponding row (a bit-slice) is returned for each. By performing a bitwise AND operation on the bit-slices, the returned column indices indicate samples containing the query k-mer. Not only is BIGSI exceptionally elegant and simple, it shows that a sketching algorithm that has been around for decades can still be adapted to create novel and high-performance genomics applications.

### Element frequency with count-min sketch

#### Element frequency

To continue with our record collection analogy, you are busy enjoying the AC/DC back catalogue but are now wondering how many times they have used that same chord progression. This is an element frequency query and, in a worst-case scenario, requires you to create a list of every chord progression used in all the songs and count the number of times each occurs.

Element frequency queries get harder when you have massive and diverse sets, where the list of counts might become too large to process or hold in memory. For example, keeping count of all k-mers observed in a genome is memory intensive. This is where the Count-Min sketch comes in.

#### Count-min sketch algorithm

The Count-Min sketch algorithm produces a sketch that is a two-dimensional matrix (*d* * *w*) which is used to approximate element frequency queries [62]. The matrix size is parameterized by two factors, epsilon and delta, where the error in answering a query is within a factor of epsilon with a probability of delta.

To add an element to a Count-Min sketch, the element is hashed by *d* pairwise independent hash functions, where each hash function maps an element to a position in the range of 1..*w*. For each position, the counter in the matrix is incremented (Algorithm 3). The requirement of hash functions to exhibit pairwise independence minimizes hash collisions. The Count-Min sketch accommodates multisets as the counters in the matrix can be incremented by values greater than one.

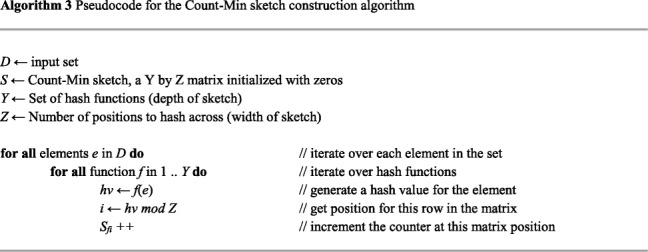



To query a Count-Min sketch and obtain a frequency estimate, an element is hashed as though it is being added to the sketch. Instead of incrementing the counter in each matrix position, the counters are evaluated and the minimum value is returned as the estimate.

#### Count-min sketch implementations for genomics

Similar to MinHash and Bloom filters, a Count-Min sketch is implemented for genomics by considering k-mers as set elements. Each k-mer is added to the sketch and the counter incremented by the k-mer frequency (Fig. 1b).

Khmer [3, 32] is a multipurpose software library for working with genomic data; at its core is a Count-Min sketch implementation for recording k-mer frequencies. Khmer also features a Bloom filter implementation for presence–absence queries. Some of the functionality of Khmer includes: read coverage normalization, read partitioning, read filtering and trimming. Count-Min Sketching is also utilized by the genome histosketching method, where k-mer spectra are represented by Count-Min sketches and the frequency estimates are utilized to populate a histosketch [4]. The Count-Min sketch has also been used for de Bruijn graph approximation during de novo genome assembly; reducing the runtime and memory overheads associated with construction of the full graph and the subsequent pruning of low-quality edges [33].

#### Considerations and variations

The Count-Min sketch is a biased estimator of element frequency, due to the possibility of counts being overestimated but not underestimated. Overestimates occur when hash collisions result in the same position in the matrix being incremented by different elements. This is mitigated by increasing the size of the sketch to reduce hash collisions, although this cannot be performed when the sketch is in use (although dynamic sketches are a possibility). Another option is to use Bayesian statistics to characterize uncertainty in the Count-Min sketch frequency approximations [63].

One variant of the Count-Min sketch involves scaling the counters during the lifetime of the sketch, allowing outdated elements to be gradually forgotten. This is an effective way of handling concept drift, whereby the distribution of the underlying data changes over time [7]. Other variants of the Count-Min sketch exist, mostly aimed at improving the performance of the sketch when it needs to be kept on disk [64].

### Set cardinality with HyperLogLog

#### Set cardinality

Suppose we want to count how many different songs you have in your record collection. You simply count all the songs by title. If you had multiple copies of a song (e.g. live recordings), you only count them once. This is a set cardinality problem (counting distinct set elements). Set cardinality problems get harder to compute when the set size grows. The classic example is counting unique website views. Counting every unique IP address to visit a website using a hash table or database needs each address to be stored, which for websites with massive traffic requires lots of memory.

#### HyperLogLog algorithm

HyperLogLog is a sketching algorithm designed to estimate the number of distinct elements in large datasets [65]. Unlike the sketch algorithms looked at so far, which use the ordering of hash values, HyperLogLog is based on bit-pattern observables. The bit-pattern observable is the number of bits until the leftmost 1 is encountered. For example, 0001010 has three leading 0 s before the leftmost 1, so the bit-pattern observable value is 4. We use bit-pattern observable probabilities to estimate set cardinality. The process is akin to flipping a coin; the odds of getting a run of two heads before tails is ½ * ½ * ½, which is  (12.5%). The odds of a run of three heads before tails is 6.25%. The odds of getting a run of *N* heads followed by a tails is 1/2^*N* + 1^. Rather than a sequence of heads or tails, think of a binary string (e.g. 0001010). The chance of seeing *N* 0 s followed by a 1 is 1/2^*N* + 1^.

The key idea behind the HyperLogLog algorithm is that by applying a uniform and deterministic hash function to get a set permutation (à la MinHash), you use bit-pattern observables of the hash values to estimate the number of unique values in the set [9]. If you encounter the hash value 0001010, which has a bit-pattern observable of 4, you can estimate you’ve seen 2^4^ distinct values. We use this logic to estimate set cardinality by finding the longest run of 0 s. If the longest is *N* 0 s and then a 1, you have probably seen around 2^*N* + 1^ elements in your set. However, because this process is stochastic (you might have one element but its hash is 000001, giving an estimated 2^6^ elements), we need to average multiple estimates.

To take multiple estimates we use the concept of registers. The HyperLogLog sketch is an array of registers; each records a count and is addressable with a unique identifier. By taking multiple estimates and then using stochastic averaging to reduce variance, HyperLogLog gives a cardinality estimate within defined error bounds.

To add an element to the sketch, it is hashed and the prefix (first *A* bits) of this value is removed and used to lookup a register in the sketch. The remainder of the hash value (the suffix) is used for the bit-pattern observable; the register is updated if the new bit-pattern observable is greater than the current one (Algorithm 4). To obtain a cardinality estimate from this sketch, the harmonic mean is calculated across all sketch registers and this is the approximate number of distinct elements in the set.

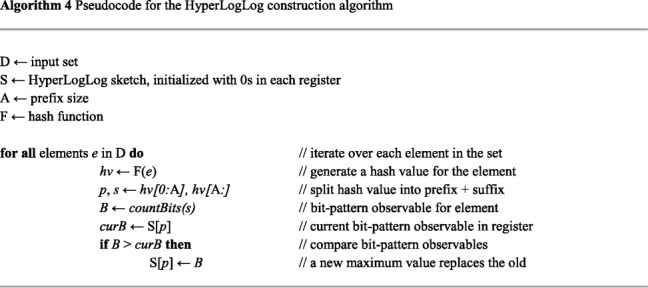



#### HyperLogLog implementations for genomics

HyperLogLog can estimate the number of distinct k-mers in a set (Fig. 1b). HyperLogLog has recently been implemented in the Dashing software for estimation of genomic distances [2]. Similar to MinHash methods, Dashing uses sketches to summarize genomes and calculates pairwise distances based on k-mer set cardinality. HyperLogLog generally results in faster sketching and greater accuracy compared to MinHash-based methods [2]. In addition, HyperLogLog does not suffer from the same performance degradation as MinHash when dealing with varying set size. However, for distance estimations HyperLogLog can be slower (compared with BinDash). Another limitation is that Dashing cannot report intersections (k-mers common between sets).

HyperLogLog is used by Khmer for counting distinct k-mers in samples [32]. It has also been used by krakenUniq for metagenome classification, specifically to approximate how many unique k-mers are covered by reads [25]. This improves upon the original classifier by enabling distinction between false-positive and true-positive database matches. The Meraculous assembler is another example of bioinformatic software that has been optimized using HyperLogLog; in this case, estimating k-mer cardinality for informing Bloom filter size [66].

#### Considerations and variations

As already mentioned, the main limitation of HyperLogLog is that it cannot accurately perform set intersection or difference operations. These operations are better suited to algorithms such as MinHash.

HyperLogLog currently has only a few implementations in genomics, with no variants that the author is aware of. In the wider data science field there are variants such as HyperLogLog++, which has an updated bias correction scheme [67], and the sliding HyperLogLog, which is designed to operate on data streams [68].

### Other algorithms

Several common set queries and the sketching algorithms designed to approximate them have now been covered. There are many more algorithms not covered which are already used in genomic research. For instance, histogram similarity using histoksetch can classify microbiomes based on incomplete data streams [4]. Another histogram-based sketch is ntCard [35], which uses a multiplicity table of hashed k-mers for estimating k-mer coverage frequencies.

The Counting Quotient Filter [60] is a sketch for approximate membership and counting queries, with space usage as good or better than CountMin sketch. The Counting Quotient Filter is used by the Mantis software to index and search sequence collections; the index being smaller and faster than a sequence bloom tree [38]. It can also be used for constructing weighted de Bruijn graphs [69].

K-mer substring space decomposition (KSSD) is a recently proposed alternative to locality sensitive hashing schemes (such as MinHash), which uses random k-mer substring space sampling to generate sketches of genomic sequences [39]. KSSD offers significant improvements (both in terms of accuracy and resource usage) over MASH screen for containment analysis but is currently restricted to use with long sequences (> 10 kbp) as shorter sequences cannot undergo KSSD dimensionality reduction and still yield an informative sketch.

The field is constantly being augmented with new algorithms and implementations. In an effort to keep a current list of sketching algorithms for genomics, please refer to the accompanying repository and file a pull request if tools are missing [40].

## Workflows for genomics

Several workflows for genomics that utilize sketching algorithms have been included in this article. These are available (see “Availability of data and material” section) and can be run interactively via Binder [8, 70]. These workflows tackle the various stages of an outbreak investigation from a paper by Reuter et al. [71], which compared whole genome sequence analysis of bacterial isolates with standard clinical microbiology practice. They demonstrated that genomics enhanced diagnostic capabilities in the context of investigating nosocomial outbreaks caused by multidrug-resistant bacteria. However, the authors relied on intensive analyses such as genome assembly and phylogenetic tree construction. Here, it is shown that sketching algorithms can replicate their analyses in several minutes, using just 1 GB of memory and not needing to store data on disk.

Workflow 1 demonstrates the use of Count-Min sketches and Bloom filters for checking and improving sequence read quality [30, 32], as well as showing how MinHash sketches can be used to classify sequence data [22]. Workflow 2 performs resistome profiling of bacterial isolates using MinHash and Minimizers while sequence data are read from online repositories [25, 28]. Workflow 3 replicates the outbreak surveillance of Reuter et al. [71] using MinHash distances to build a Newick tree that shared the same topology as the phylogeny from the paper [1, 26]. Workflow 4 augments the analysis from the original paper by using the Bloom filter-based BIGSI to identify additional isolates matching the resistome profile of the outbreak bacteria [31].

## Conclusions and future directions

Sketching clearly offers many benefits for genomic research. It has been shown how sketches can compress, index and search data, using a fraction of the resources needed by other classes of algorithms. Sketching algorithms are therefore a great approach for processing huge amounts of genomic data while using basic hardware (e.g. laptops). Sketching also has applications for data privacy, whereby sketching effectively anonymizes confidential data and enables remote analytics. For example, Balaur is a read aligner that sketches data to preserve privacy before outsourcing alignments to cloud compute [72].

Many exciting genomics applications for sketching are beginning to be realized. As sketches are relatively stable and very compact, they are excellent candidates for database indexing and compression. This functionality is already being used by projects such as sourmash, which are able to provide indexed collections of reference sequences that are ready to be interrogated using user-defined sketches [22]. This could allow you to download any genome collection, sketching the download in real-time and using this information to inform downstream analysis, e.g. what genomes to write to disk or to analyze on HPC. This real-time usability of sketches lends them to machine learning applications. We recently showed their utility as succinct representations of microbiome data streams that can be used to predict information about the samples [4, 73]. Sketching has clear potential in real-time analytics, such as for monitoring sequencing progress.

In response to the recent adoption of sketching algorithms for genomics, this review has set out to cover how these algorithms can be used to address some of the challenges we are encountering as genomic data sources continue to grow. Hopefully it has provided an understanding of how sketching algorithms work, their benefits and limitations and how sketching can be applied to existing genomic workflows. If you wish to continue reading more on the topic of sketching algorithms, the excellent review that was recently published by Marçais et al. [74] is recommended.

## Supplementary information

Additional file 1: Review history.(DOCX 25 kb)

## Data Availability

The source code for the notebooks is available under an MIT License (at https://github.com/will-rowe/genome-sketching) and the live notebooks are available via Binder [8, 70]. The genome data used in the notebooks is available from the ENA under BioProject PRJEB3353 [74].
