# Supplementary material for: When the levee breaks: a practical guide to sketching algorithms for processing the flood of genomic data
Source: Genome Biol. 2019 Sep 13;20:199. doi: 10.1186/s13059-019-1809-x (PMC6744645; doi:10.1186/s13059-019-1809-x)
Supplement: Supplementary file 1 — Review history.(DOCX 25 kb) [file 13059_2019_1809_MOESM1_ESM.docx]

Review History

**First round of review**

**Reviewer 1**

**Is the topic of the article timely and of interest to a wide range of readers?**

Yes

**Is the article well written and presented in a logical way?**

Yes

**Do the authors cover the relevant literature in an accurate and balanced way?**

Yes

**Do the authors provide a useful synthesis of the topic?**

Yes

**Do the authors provide insightful discussion of the future directions for the field?**

No

**Do any tables, figures or boxes that are present enhance the manuscript?**

Yes

**Comments to author:**

Rowe provides a light review of sketching algorithms applied to genomic data. The topic is relevant and of rising interest in bioinformatics. Although not mentioned in the title or abstract, the focus is specifically on microbial genome data and no mention is made of applications in other areas of genomics where these methods have been applied, such as human transcriptomics. It would have been nice to see a broader scope. It is also a somewhat shallow review that would serve as a basic introduction of sketching concepts for microbial bioinformaticians. The associated Python workbooks are a nice touch. The writing style of the review is informal with frequent use of the first and second person, making it read like a blog post, but I'm not opposed to this. The language is accessible although not very rigorous. I am just left wondering what readers will take away from this piece. As a methods developer, it didn't teach me anything new. For those that are not already familiar with these techniques, it is not deep enough to grant a solid understanding but does give some helpful pointers to the relevant literature. That said, the accuracy of the piece is fine and I did not see anything completely objectionable. So, many of the following comments should be taken only as my opinions on how the piece could be improved. In rough order as appearing in the text:

- The phrase "sketching algorithms" is used in the abstract without ever being defined. If a reader only reads the abstract, will they be able to tell what the piece is about?

- The purpose of Figure 1 is unclear. I do not think it adds to the paper.

- The string and set processing analogies jump between English text, collections of music records, and microbial genomics. I didn't really see the need for this and thought the record collection analogies were distracting.

- A major strength of the sketching algorithms is for estimating averages, e.g. the average similarity of two genomes. In these cases it is unnecessary to examine every last k-mer to get a good measure of similarity. I thought this point should be made somewhere in the piece.

- "...if the same hash function is applied to two sets, the chance of both permutations having the same..." The permutation is defined on the set of all k-mers, so there is only one permutation in this case. Should say "the chance of both sets" instead.

- "is going to be equal to the ratio of the number of common elements to the size of the union, i.e. the Jaccard similarity" is stated as an unreferenced fact, but this is not immediately obvious. Would be better to say something to the effect of "Broder proved that..."

- In Figure2a in the HyperLogLog example, a single bit is indicating the register in the 2nd column, but three registers are shown in the 3rd column. How can one bit address three registers? Also, in the associated caption it says the observable bit pattern is greater than the current one, when it is in fact less than. I'd suggest talking about leading zero's rather than greater/less to avoid endianness confusion.

- Although Mash is perhaps the most well known, it was not the first MinHash implementation for genomic data. See prior use of MinHash in other genomic contexts, e.g. MHAP (Berlin), Mc-minh (Rasheed).

- Table 2, some missing tools that use related sketching ideas: MHAP (Berlin), KmerStream (Melsted), BFcounter (Melsted), KmerGenie (Chikhi), Squeakr (Pandey), Mantis (Pandey), Sequence Bloom Tree (Solomon), etc. How were the tools shown chosen? It would be nice if the table was as comprehensive as possible. That would be a valuable contribution of this review. Also, MashMap is listed as a bottom-k method, but it uses minimizers as the primary indexing scheme (like MiniMap).

- "Of note are sourmash, which uses cosine similarity for weighted similarity estimates". Sourmash does this incorrectly, so I would not highlight this feature. It apparently computes cosine similarity on top of MinHash sketches, which is mathematically nonsensical. Cosine similarity cannot be estimated via MinHashing and would require using an alternative sketching method such as SimHash to properly estimate.

- Algorithm pseudocode captions should read "Bloom filter *construction* algorithm" and "Count-Min sketch *construction* algorithm" etc. since these are names of data structures, not algorithms.

- Reference [28] appears wrong. It appears in the text after mention of "BFCounter", but the reference points to the "khmer" software. BFCounter is by Melsted and Pritchard, and does not appear anywhere in the references (but should).

- The text credits "khmer" as "one of the earliest implementations of HyperLogLog", but the Georganas paper predates it by a year. Why give more credit to the later paper?

- Section 4 should lead with a URL pointing to the workflows. Each of the workflows should list its dependancies and include links to the tools used.

- bbtools (sf.net/projects/bbmap) has a nicer interface for querying a sketch database than mash and sourmash. It queries a remote sketch database and so does not require downloading a sketch database like the other two tools. Should be mentioned.

- I could not find reference [59]. Is it available anywhere?

- Some terms are bolded but not included in the definitions table, e.g. "minimizers", "k-mer decomposition", "cosine similiarity", "Stochastic averaging", etc.

A few quibbles over definitions:

- "Canonical k-mers" are defined as being the lexicographically smallest of a k-mer and its reverse complement. It is not necessary to define it this way. For example, it could be defined as the smallest hash value rather than lexicographically.

- "Jaccard similarity" is defined as "a similarity measure defined as the intersection of sets, divided by their union". This definition should specify that it is the cardinality of these sets that is being measured.

- "Sketch" is defined as having a "fixed size". This is not always true. For example, windowed minimizer sketching results in a sketch size that grows linearly with the input size.

- "Weighted set" is defined as "a set that allows for multiple instances of each of its elements". This would be a "multiset". In a weighted set, the elements could be arbitrarily weighted, not just by frequency.

- "Hash functions take large input data of arbitrary size and map it to a much smaller bit string" This distinction between size of input and hash value is not true for k-mers (the most common use in this context). The number of bits needed for a k-mer and its corresponding hash value are typically about equal.

**Reviewer 2**

**Is the topic of the article timely and of interest to a wide range of readers?**

Yes

**Is the article well written and presented in a logical way?**

Yes

**Do the authors cover the relevant literature in an accurate and balanced way?**

Yes

**Do the authors provide a useful synthesis of the topic?**

Yes

**Do the authors provide insightful discussion of the future directions for the field?**

No

**Do any tables, figures or boxes that are present enhance the manuscript?**

Yes

**Comments to author:**

The author reviews sketching methods used to summarize large data sets using reduced representations. The manuscript is written clearly using accessible phrasing.

Please add page numbers to the manuscript.

My best regards,

Shaun Jackman

\# Major comments for revision

I believe the "MinHash KMV sketch" described should instead be called a "k-mins sketch" (see MinHash Sketches: A Brief Survey, Edith Cohen, 2016). KMV is a different algorithm for the purpose of estimating the frequency moments (such as the F_0 cardinality) of a data set. See https://en.wikipedia.org/wiki/Streaming_algorithm#K-Minimum_Value_Algorithm

Is there a specific reference for the variant of the k-mins sketch described in algorithm 1 that uses exactly two hash functions and "hv <- hv_1 + (i * hv_2)"? Is this equation missing a modulus operator?

> Algorithm 3

The algorithm here describes a counting Bloom filter, not a count min sketch. In particular the data structure of a counting Bloom filter is a vector, whereas for a count min sketch it is a matrix of size D by W. For a count min sketch, there is one row for each hash function. For a counting bloom filter, there is only one row shared by all hash functions.

> X <- Count-Min sketch, initialised with 0s in each position

Change to "X <- a D by W matrix initialized with zeros"

> i <- hf % w

Use "mod" rather than "%".

> X_i++

Should be "X_fi <- X_fi + 1". Note the "f" which indexes the row.

\# Minor comments for revision

> sketch: a fixed-size, compact data structure that approximates a data set

Not all sketches are fixed size. A vector of minimizers is an example of a sketch that does not have fixed size and varies with the length of the input sequence.

> Standard error of a sample of size s is 1 / √s , whereas sketches of size s can guarantee error that is proportional to 1 / s [5].

Please typeset these equations and square root symbol.

> Each collection is a set and we can use Jaccard similarity, defined as the intersection of two sets divided by their union, to measure their similarity.

Please change to "Each collection is a set and we can use Jaccard similarity, defined as the size of the intersection of two sets divided by the size of their union, to measure their similarity."

> If our two collections have 60 records in common, then the Jaccard similarity is 60 divided by the number of unique records 140, giving 0.429.

I prefer the term "distinct" in this context rather than "unique".

> Document similarity can be based simply on the number of shared words, but to take into account document structure, it may be better to represent the document as a set of overlapping groups of words.

It may be helpful in this section to describe the relevant terms "bag of words model" and "n-gram".

> The Jaccard similarity is 0.176 if structure is not important, but 0 if it is.

Consider "The Jaccard similarity is 0.176 using a bag of words model, but 0 using a bag of 6-word-grams model, which retains some structure."

> So, if we want to perform set similarity queries

Consider "To perform set similarity queries" striking "So, if we want".

> the chance of both permutations having the same lowest hash value

Consider "minimal" rather than "lowest".

> The MinHash sketch data structure is just a set of hash values

"The MinHash sketch data structure is a vector of hash values". A vector preserves the order of the elements, whereas a set does not. The order of the hash values is important to a MinHash sketch.

> Table 2

Please add ntCard to this table.

> Algorithm 2

Use "mod" rather than "%".

> To calibrate a Bloom filter, the false positive rate is proportional to the sketch length

"To calibrate a Bloom filter, the false positive rate is inversely proportional to the sketch length"

> The more hash functions a Bloom filter uses the slower it will be, but using too few functions will result in more false positives.

Too many hash functions also increases the FPR. Consider "The more hash functions a Bloom filter uses the slower it will be, but using too few functions or too many will result in more false positives. A simple calculation computes the optimal number of hash functions for a given size of Bloom filter, but requires knowing and an estimate of the number of distinct elements." The formula could be given if the authors wish. It's available from https://en.wikipedia.org/wiki/Bloom_filter#Optimal_number_of_hash_functions

> Bloom filters are also used in conjunction with other sketching algorithms, such as by MASH to prevent unique k-mers

I prefer the term "singleton" k-mers rather than "unique" in this context. The term "unique" may be confused with "distinct".

> However, because this process is random

Use "stochastic" rather than "random"

> As sketches are relatively stable, a genome sketch is unlikely to change if an assembly is updated

If the updated assembly changes the sequences of the assembly which changes the k-mers present in the assembly, the genome sketch is likely to change. In fact a genome sketch would not be very useful if it did not change when the genome changes.

**Authors Response**

**Point-by-point responses to the reviewers’ comments:**

General comment: I wish to thank the reviewers for their excellent comments and suggestions. They were fair and constructive, resulting in what I feel is a much improved manuscript. Thank you for taking the time to read the paper and provide such detailed feedback.

Reviewer #1:

Rowe provides a light review of sketching algorithms applied to genomic data. The topic is relevant and of rising interest in bioinformatics. Although not mentioned in the title or abstract, the focus is specifically on microbial genome data and no mention is made of applications in other areas of genomics where these methods have been applied, such as human transcriptomics. It would have been nice to see a broader scope. It is also a somewhat shallow review that would serve as a basic introduction of sketching concepts for microbial bioinformaticians. The associated Python workbooks are a nice touch. The writing style of the review is informal with frequent use of the first and second person, making it read like a blog post, but I'm not opposed to this. The language is accessible although not very rigorous. I am just left wondering what readers will take away from this piece. As a methods developer, it didn't teach me anything new. For those that are not already familiar with these techniques, it is not deep enough to grant a solid understanding but does give some helpful pointers to the relevant literature. That said, the accuracy of the piece is fine and I did not see anything completely objectionable. So, many of the following comments should be taken only as my opinions on how the piece could be improved. In rough order as appearing in the text:

- The phrase "sketching algorithms" is used in the abstract without ever being defined. If a reader only reads the abstract, will they be able to tell what the piece is about?

*Response: This was an oversight and has since been added (ln: 11-12).*

- The purpose of Figure 1 is unclear. I do not think it adds to the paper.

*Response: Figure 1 has now been removed.*

- The string and set processing analogies jump between English text, collections of music records, and microbial genomics. I didn't really see the need for this and thought the record collection analogies were distracting.

*Response: I tried to make the introduction to each section both accessible and continuous, so that those readers new to set theory and sketching may find these topics easier to understand. Some other initial readers have found this helpful but I can see how these section introductions could also be distracting to other readers. I would personally like to leave them in but I am happy to take editorial advice on this.*

- A major strength of the sketching algorithms is for estimating averages, e.g. the average similarity of two genomes. In these cases it is unnecessary to examine every last k-mer to get a good measure of similarity. I thought this point should be made somewhere in the piece.

*Response: I agree and have now made this point explicitly in the introduction (ln: 52-55).*

- "...if the same hash function is applied to two sets, the chance of both permutations having the same..." The permutation is defined on the set of all k-mers, so there is only one permutation in this case. Should say "the chance of both sets" instead.

*Response: This line has now been changed.*

- "is going to be equal to the ratio of the number of common elements to the size of the union, i.e. the Jaccard similarity" is stated as an unreferenced fact, but this is not immediately obvious. Would be better to say something to the effect of "Broder proved that..."

*Response: Thank you for this point, I have now updated the wording and referenced the fact correctly.*

- In Figure2a in the HyperLogLog example, a single bit is indicating the register in the 2nd column, but three registers are shown in the 3rd column. How can one bit address three registers? Also, in the associated caption it says the observable bit pattern is greater than the current one, when it is in fact less than. I'd suggest talking about leading zero's rather than greater/less to avoid endianness confusion.

*Response: Thank you for catching this mistake, you are indeed right that 1 bit can’t address three registers. I have removed one of the registers from the figure. Thank you also for the suggestion regarding leading zero’s, this makes the explanation much clearer in my opinion. I have updated the caption and Figure 2a in response to this comment.*

- Although Mash is perhaps the most well known, it was not the first MinHash implementation for genomic data. See prior use of MinHash in other genomic contexts, e.g. MHAP (Berlin), Mc-minh (Rasheed).

*Response: I apologise for this oversight and have now added these.*

- Table 2, some missing tools that use related sketching ideas: MHAP (Berlin), KmerStream (Melsted), BFcounter (Melsted), KmerGenie (Chikhi), Squeakr (Pandey), Mantis (Pandey), Sequence Bloom Tree (Solomon), etc. How were the tools shown chosen? It would be nice if the table was as comprehensive as possible. That would be a valuable contribution of this review. Also, MashMap is listed as a bottom-k method, but it uses minimizers as the primary indexing scheme (like MiniMap).

*Response: Thank you for the additions, these are now in the updated Table 2. I agree that this table should be as comprehensive as possible - it is also being updated in the associated GitHub repository and I intend to do this beyond the publication of this paper.*

- "Of note are sourmash, which uses cosine similarity for weighted similarity estimates". Sourmash does this incorrectly, so I would not highlight this feature. It apparently computes cosine similarity on top of MinHash sketches, which is mathematically nonsensical. Cosine similarity cannot be estimated via MinHashing and would require using an alternative sketching method such as SimHash to properly estimate.

*Response: This is a good point; I have removed reference to the cosine similarity calculation of sourmash.*

- Algorithm pseudocode captions should read "Bloom filter *construction* algorithm" and "Count-Min sketch *construction* algorithm" etc. since these are names of data structures, not algorithms.

*Response: These captions have been updated*

- Reference [28] appears wrong. It appears in the text after mention of "BFCounter", but the reference points to the "khmer" software. BFCounter is by Melsted and Pritchard, and does not appear anywhere in the references (but should).

*Response: Apologies for this referencing error, it has now been fixed.*

- The text credits "khmer" as "one of the earliest implementations of HyperLogLog", but the Georganas paper predates it by a year. Why give more credit to the later paper?

*Response: Agreed, this has now been removed.*

- Section 4 should lead with a URL pointing to the workflows. Each of the workflows should list its dependancies and include links to the tools used.

*Response: This is a good suggestion and has been incorporated in section 4. The workflows have also been updated to link to each of the tools used as suggested.*

- bbtools (sf.net/projects/bbmap) has a nicer interface for querying a sketch database than mash and sourmash. It queries a remote sketch database and so does not require downloading a sketch database like the other two tools. Should be mentioned.

*Response: Thank you for highlighting this feature of bbsketch - I did not realise it used a remote database. That is indeed a nice feature and I have updated the paper to add this.*

- I could not find reference [59]. Is it available anywhere?

*Response: I have chased this up and the conference proceedings are still in press, I have now added the in press tag to the citation.*

- Some terms are bolded but not included in the definitions table, e.g. "minimizers", "k-mer decomposition", "cosine similiarity", "Stochastic averaging", etc.

*Response: The definitions table has now been updated.*

A few quibbles over definitions:

- "Canonical k-mers" are defined as being the lexicographically smallest of a k-mer and its reverse complement. It is not necessary to define it this way. For example, it could be defined as the smallest hash value rather than lexicographically.

*Response: This has now been changed.*

- "Jaccard similarity" is defined as "a similarity measure defined as the intersection of sets, divided by their union". This definition should specify that it is the cardinality of these sets that is being measured.

*Response: This has now been changed.*

- "Sketch" is defined as having a "fixed size". This is not always true. For example, windowed minimizer sketching results in a sketch size that grows linearly with the input size.

*Response: This has now been changed.*

- "Weighted set" is defined as "a set that allows for multiple instances of each of its elements". This would be a "multiset". In a weighted set, the elements could be arbitrarily weighted, not just by frequency.

*Response: This has now been changed.*

- "Hash functions take large input data of arbitrary size and map it to a much smaller bit string" This distinction between size of input and hash value is not true for k-mers (the most common use in this context). The number of bits needed for a k-mer and its corresponding hash value are typically about equal.

*Response: This has now been changed.*

Reviewer #2: The author reviews sketching methods used to summarize large data sets using reduced representations. The manuscript is written clearly using accessible phrasing.

Please add page numbers to the manuscript.

My best regards,

Shaun Jackman

\# Major comments for revision

I believe the "MinHash KMV sketch" described should instead be called a "k-mins sketch" (see MinHash Sketches: A Brief Survey, Edith Cohen, 2016). KMV is a different algorithm for the purpose of estimating the frequency moments (such as the F_0 cardinality) of a data set. See https://en.wikipedia.org/wiki/Streaming_algorithm#K-Minimum_Value_Algorithm

*Response: I apologise for my confusion here and have updated the manuscript with more accurate and consistent terminology. I have actually seen several conflicting definitions of KMV MinHash and your comment prompted me to read into this further. I think that my confusion has stemmed from the K-minimum value streaming algorithm and the K-minimum values variant of MinHash. I now have elected to use the terminology for MinHash variants that was used in the HyperMinHash paper (Yu et al.), which are:*

*K-hash functions*

*K-min values (synonymous with bottom-k)*

*K-partition*

*I feel these are more consistent and more intuitive. Please let me know if you disagree. I have provided references for the naming of each of these sketches.*

Is there a specific reference for the variant of the k-mins sketch described in algorithm 1 that uses exactly two hash functions and "hv <- hv_1 + (i * hv_2)"? Is this equation missing a modulus operator?

*Response: I was basing this on implementations of K-hash MinHash I have come across in MinHash libraries that save on hash functions by mapping one or two functions to the desired number of permutations. (e.g. https://github.com/dgryski/go-minhash). However, I thought it might now be better to simplify this part completely and I’ve elected to use a set of k hash functions instead to illustrate the KHF concept. I have now updated this algorithm pseudocode.*

Algorithm 3

The algorithm here describes a counting Bloom filter, not a count min sketch. In particular the data structure of a counting Bloom filter is a vector, whereas for a count min sketch it is a matrix of size D by W. For a count min sketch, there is one row for each hash function. For a counting bloom filter, there is only one row shared by all hash functions.

X <- Count-Min sketch, initialised with 0s in each position

Change to "X <- a D by W matrix initialized with zeros"

i <- hf % w

Use "mod" rather than "%".

X_i++

Should be "X_fi <- X_fi + 1". Note the "f" which indexes the row.

*Response: Thank you for pointing this out, I apologise for my mistake here and I have now corrected this pseudocode.*

\# Minor comments for revision

sketch: a fixed-size, compact data structure that approximates a data set

Not all sketches are fixed size. A vector of minimizers is an example of a sketch that does not have fixed size and varies with the length of the input sequence.

*Response: This has now been changed.*

Standard error of a sample of size s is 1 / √s , whereas sketches of size s can guarantee error that is proportional to 1 / s [5].

Please typeset these equations and square root symbol.

*Response: This has now been changed.*

Each collection is a set and we can use Jaccard similarity, defined as the intersection of two sets divided by their union, to measure their similarity.

Please change to "Each collection is a set and we can use Jaccard similarity, defined as the size of the intersection of two sets divided by the size of their union, to measure their similarity."

*Response: This has now been changed.*

If our two collections have 60 records in common, then the Jaccard similarity is 60 divided by the number of unique records 140, giving 0.429.

I prefer the term "distinct" in this context rather than "unique".

*Response: This has now been changed.*

Document similarity can be based simply on the number of shared words, but to take into account document structure, it may be better to represent the document as a set of overlapping groups of words.

It may be helpful in this section to describe the relevant terms "bag of words model" and "n-gram".

*Response: Agreed - I’ve now added these terms.*

The Jaccard similarity is 0.176 if structure is not important, but 0 if it is.

Consider "The Jaccard similarity is 0.176 using a bag of words model, but 0 using a bag of 6-word-grams model, which retains some structure."

*Response: This sentence has been updated to reflect the new terms.*

So, if we want to perform set similarity queries

Consider "To perform set similarity queries" striking "So, if we want".

*Response: This has now been changed.*

the chance of both permutations having the same lowest hash value

Consider "minimal" rather than "lowest".

*Response: This has now been changed.*

The MinHash sketch data structure is just a set of hash values

"The MinHash sketch data structure is a vector of hash values". A vector preserves the order of the elements, whereas a set does not. The order of the hash values is important to a MinHash sketch.

*Response: This has now been changed.*

Table 2

Please add ntCard to this table.

*Response: This has now been changed.*

Algorithm 2

Use "mod" rather than "%".

*Response: This has now been changed.*

To calibrate a Bloom filter, the false positive rate is proportional to the sketch length

"To calibrate a Bloom filter, the false positive rate is inversely proportional to the sketch length"

*Response: This has now been changed.*

The more hash functions a Bloom filter uses the slower it will be, but using too few functions will result in more false positives.

Too many hash functions also increases the FPR. Consider "The more hash functions a Bloom filter uses the slower it will be, but using too few functions or too many will result in more false positives. A simple calculation computes the optimal number of hash functions for a given size of Bloom filter, but requires knowing and an estimate of the number of distinct elements." The formula could be given if the authors wish. It's available from https://en.wikipedia.org/wiki/Bloom_filter#Optimal_number_of_hash_functions

*Response: This is a good suggestion and has now been added.*

Bloom filters are also used in conjunction with other sketching algorithms, such as by MASH to prevent unique k-mers

I prefer the term "singleton" k-mers rather than "unique" in this context. The term "unique" may be confused with "distinct".

*Response: This has now been changed.*

However, because this process is random

Use "stochastic" rather than "random"

*Response: This has now been changed.*

As sketches are relatively stable, a genome sketch is unlikely to change if an assembly is updated

If the updated assembly changes the sequences of the assembly which changes the k-mers present in the assembly, the genome sketch is likely to change. In fact a genome sketch would not be very useful if it did not change when the genome changes.

*Response: This is a good point, and one I should have thought through more thoroughly. I’ve removed this statement now.*

**Second round of review**

**Reviewer 1**

The author has sufficiently addressed my prior comments, and the revised manuscript is an improvement over the initial version. The manuscript is still heavily focused on microbial genomics, but this is forgivable for the sake of brevity. A truly comprehensive review of this topic, considering all possible applications, is a nearly impossible task.

A few lingering issues I spotted in the revision:

Table 1: "a cardinality measure defined as the intersection of sets, divided by their union". I wouldn't call the Jaccard itself a "cardinality" measure. It operates on the cardinalities of the intersection and union, but is not itself a cardinality measure.

Section 3.1.1. "defined as the intersection of two sets divided by the size of their union" replace with "defined as the size of the intersection of two sets, divided by the size of their union."

Section 3.1.2. "there is a performance cost associated with sorting the sketch prior to estimating Jaccard similarity between sketches [16]." We recently discussed reference [16] in a journal club and I believe this statement is an error in the preprint. KMV sketch generation is O(n log k), and if the priority queue is implemented as a balanced binary tree, the resulting sketch is already in sorted order when it is output. Thus, comparing or merging the sorted sketch with another is an O(k) time merge operation. So I do not understand where this additional cost the authors mention is coming from.

Figure 1a. I was confused when I first looked at this figure because I thought that the "C" and "G" characters were missing from the 1st and 2nd lines, when instead the k-mers were wrapping around. It might be clearer to just add those two extra k-mers to the bottom of the figure to avoid that illusion.

Section 3.1.4. "enabling calculation of edit distance between sequences [43]" I don't believe Order MinHash is actually capable of "calculating edit distance". This paper only shows that it is a (s1, s2, p1, p2)-sensitive LSH scheme.

"A key property of sketches that I've not covered is that they are mergable" This is not true of all sketches, is it? I recall the HyperMinHash paper making the point that certain types of sketches (e.g. b-bit MinHash) are *not* mergable, and this is why the HyperMinHash scheme was preferred over the other variants.

**Reviewer 2**

The authors have addressed my previous concerns with their revised manuscript.

**Authors Response**

**Point-by-point responses to the reviewers’ comments:**

General comment:

*I wish to thank the reviewers and editor again for their thorough review of the manuscript. My apologies for the delay in my response - I was on annual leave. I have now addressed the remaining comments and issues. Since the original submission, I have moved institutes and I have now updated my contact information to reflect this.*

Editorial comments:

*I have added a few additional, non-microbial, sketching examples.*

*The github repository for this manuscript is now correctly referenced.*

*I have now made the suggested updates to the title and abstract.*

*All figure legends are now after the references section.*

*Highlighted text has been removed.*

*Main figures are now removed from the main text and supplied as individual files.*

Reviewer 1:

*The definition of Jaccard similarity in Table 1 has been updated to state it is a “similarity measure”.*

*The definition of Jaccard similarity in 3.1.1 has been updated as suggested.*

*The erroneous statement in 3.1.2 has been removed.*

*Figure 1a has been updated to improve readability*

*The statement regarding Order MinHash has been updated to state that it provides an approximation of sequence edit distance*

*The statement regarding mergability has been removed from the conclusion.*

Reviewer 2:

*I have now added a sentence to the conclusion section, directing readers to the suggested paper for further reading.*
